# Strategies in activating lymphatic system to promote lymph flow on lymphedema symptoms in breast cancer survivors: A randomized controlled trial

**DOI:** 10.3389/fonc.2022.1015387

**Published:** 2022-10-24

**Authors:** Xinwen Du, Yuan Li, Lan Fu, Huaying Chen, Xiaoxia Zhang, Yuping Shui, Aihua Zhang, Xianqiong Feng, Mei Rosemary Fu

**Affiliations:** ^1^ Department of Hematology, West China Hospital/West China School of Nursing, Sichuan University, Chengdu, China; ^2^ Department of Nursing, West China Second University Hospital/West China School of Nursing, Sichuan University, Chengdu, China; ^3^ Cancer Center, West China Hospital/West China School of Nursing, Sichuan University, Chengdu, China; ^4^ Head & Neck Oncology Ward, Cancer Center, West China Hospital/West China School of Nursing, Sichuan University, Chengdu, China; ^5^ Department of Operating Room, West China Hospital/West China School of Nursing, Sichuan University, Chengdu, China; ^6^ West China School of Nursing, Sichuan University, Chengdu, China; ^7^ School of Nursing–Camden, Rutgers, The State University of New Jersey, Camden, NJ, United States

**Keywords:** breast cancer survivors, lymphedema, lymphatic system, early intervention, lymphatic exercises, mobile health, randomized controlled trial

## Abstract

**Background:**

Many breast cancer survivors face long-term postoperative challenges as a result of developing lymphedema symptoms and chronic lymphedema. *The-Optimal-Lymph-Flow* (TOLF) program is an intervention based on physiological-cognitive-behavioral principles that teaches patients self-management strategies to activate lymphatic system and promote lymph flow to decrease lymphatic pain, reduce the risk and severity of lymphedema.

**Objective:**

The purpose of this pilot clinical trial was to evaluate the use of TOLF program as an early intervention on improving lymphedema symptom experience (i.e., symptom number, symptom severity, symptom distress, and the impact of symptoms on patients’ activities of daily living) and optimizing lymph fluid levels (measured by the arm volume differences) among breast cancer survivors.

**Methods:**

This study is a parallel, randomized clinical trial. A total of 92 breast cancer patients were randomly assigned to either the TOLF intervention group or the control group focusing on promoting arm mobility. Data were collected at baseline and end of the trial at the 3-month post intervention. The Breast Cancer and Lymphedema Symptom Experience Index was used to measure lymphedema symptom experience. Anthropometric measurements were used for circumferential arm measurements. Generalized linear mixed-effects models were used to evaluate the trial outcomes.

**Results:**

Significant improvements of lymphedema symptom experience were found in patients in the TOLF intervention group in comparison with patients in control group: the number of lymphedema symptoms (*P*<0.001) and the severity of lymphedema symptoms (*P*<0.001) as well as the impact of symptoms on patients’ daily living function (*P*<0.001). Patients in both groups showed improvements in all study outcomes over the 3 months, whereas those in the TOLF group gained greater benefits in reducing the number and severity of lymphedema symptoms. Moreover, the TOLF group had significantly fewer patients with ≥5% arm volume differences ([5/45] vs [13/43], *P*=0.035) at the study endpoint.

**Conclusions:**

Findings of the study demonstrated positive outcomes of relieving lymphedema symptom experience, optimizing arm circumference and halting the progression of lymphedema status in breast cancer survivors receiving TOLF intervention during early postoperative time. Given its feasibility, acceptability, and effectiveness, this program may be incorporated in routine breast cancer care.

**Clinical Trial Registration:**

http://www.chictr.org.cn/index.aspx, identifier ChiCTR1800016713.

## Introduction

Breast cancer is the most common malignancy in women worldwide, with 2.3 million new cases diagnosed annually, and due to improvements in early detection and treatment, the population of breast cancer survivors is constantly expanding (1). Lymphedema is a prevalent and debilitating complication of breast cancer treatment affecting more than 1 in 5 breast cancer survivors (2, 3), leading to chronic ipsilateral arm swelling coupled with multiple distressing symptoms (e.g., swelling, pain, heaviness, tightness, firmness, numbness, stiffness, or impaired arm mobility) due to abnormal fluid accumulation defined as lymphedema symptoms (4, 5). It is well documented that lymphedema and associated symptoms are perceived as constant reminders of cancer and exert tremendous limitations on patients’ daily living function and quality of life (6, 7).

In fact, the latent stage of lymphedema may exist months or years before the overt lymphedema occurs (2). Without timely detection and intervention in the early stage, lymphedema can progress into a chronic condition that no surgical or medical interventions can cure (8, 9). Lymphedema symptoms, experienced by over 50% of women treated for breast cancer, are significant predictors of lymphedema (10, 11). The experience of lymphedema symptoms for breast cancer patients without a diagnosis of lymphedema is a cardinal sign of an early stage of lymphedema because these symptoms often precede changes in arm girth and a lymphedema diagnosis (12–14). Breast cancer survivors who reported 5 or more lymphedema symptoms are more likely to develop lymphedema (15). Effective management of lymphedema symptoms can decrease the risk of developing lymphedema (16–19). Thus, managing lymphedema symptoms is imperative to prevent lymphedema, maintain a normal arm volume, as well as improve activities of daily living (ADLs).

Very limited studies have been designed to build patients’ self-management skills to manage lymphedema symptoms, which are major predictors for lymphedema, impaired physical function, psychological distress, and poor quality of life (11, 20, 21). Majority of previous randomized controlled trials (RCT) were devoted to lymphedema treatment administered by professional therapists (22, 23). However, cost and time to attend therapist-administered treatments remain wide-spread challenges for patients. *The-Optimal-Lymph-Flow* (TOLF) program is an intervention based on physiological-cognitive-behavioral principles that teaches patients self-management strategies to activate lymphatic system and promote lymph flow to decrease lymphatic pain, reduce the risk and severity of lymphedema (24, 25). TOLF features a web- and mobile-based mHealth system that includes information about lymphedema knowledge and self-care strategies to deliver safe, easy, and feasible digital therapy of lymphatic exercises (i.e., muscle tightening–breathing, muscle tightening–pumping exercises, large muscle exercises) to promote lymph flow and drainage, limb mobility exercises to enhance shoulder and arm function, and general instructions to encourage healthy weight and proper sleep (16, 18, 24, 25). The core TOLF intervention is the 8-minute TOLF lymphatic exercises (16). Patients can learn and follow all the therapeutic exercises through avatar video simulations by logging onto the TOLF system anywhere and anytime with a computer, laptop, or any mobile phones or tablets (17, 25). The effectiveness of TOLF intervention has been demonstrated in several trials (17–19, 25). However, TOLF has not been tested as an early intervention to mitigate lymphedema symptoms, optimize ADLs, and prevent lymphedema. This pilot randomized clinical trial (RCT) was designed to evaluate the use of TOLF program as an early intervention for patients at risk for lymphedema on improving lymphedema symptom experience (i.e., symptom number, symptom severity, symptom distress, and the impact of symptoms on patients’ ADLs and optimizing lymph fluid levels among breast cancer survivors.

## Materials and methods

### Study design

This was a prospective RCT conducted from January 2019 to June 2020. Potential participants were screened for the risk of lymphedema at 1 month after surgical treatment and those reported 5 or more lymphedema symptoms were deemed as at-risk patients; subsequently and randomly assigned to either the TOLF intervention group or arm mobility control group to assess the effects of TOLF program on lymphedema symptom experience and lymph fluid levels. This trial was registered at the Chinese Clinical Trial Registry (ChiCTR1800016713). The protocol was in accordance with the CONSORT-EHEALTH checklist (26) (**Supplementary Material**).

### Participants and setting

The study was conducted in West China Hospital, a 4300-bed tertiary teaching hospital affiliated with Sichuan university and the leading medical center in the south-western China. Breast cancer patients were introduced to the trial when attending their routine follow-up visits at 1 month after the surgical treatment. Patients were included if they (1) were nonpregnant females aged 18 to 80 years (2); underwent surgical treatment for breast cancer for the first time (3); reported at least 5 lymphedema symptoms (4); had not been diagnosed with lymphedema (5); had access to a smartphone, tablet device or computer/laptop with Internet access; and (6) willing to follow the self-management strategies. The exclusion criteria were as follows (1): patients who had a delayed healing of the incisions (2); the presence of local and distant metastasis (3); patients with history of surgery or trauma on the affected axilla or arm (4); patients with severe mental illness or cognitive impairments (5); patients who had documented advanced cardiac or renal diseases; or (6) with non-breast-cancer-related lymphedema. Each participant signed the written study consent.

### Randomization

This study used computer-generated random numbers for randomization with a 1:1 ratio. The random numbers were concealed and administered by the project coordinator. Eligible patients were randomly allocated to either the TOLF intervention group or the arm mobility control group. The interventionists were blinded to group assignment. Participants, outcome assessors, and statistician were unaware which treatment was the intervention of interest and which one was the comparator.

### Intervention

The intervention strategies for the two groups are presented in **Table 1**. Patients assigned to the TOLF intervention group were granted full access to the web- and mobile-based TOLF platform to learn about all the involved contents. They had the access to the Lymphedema Knowledge module to learn about the lymphatic system, lymphedema basics (e.g., definition, prevalence, diagnosis, symptoms, treatment and etc.), self-care skills, and general instructions about keeping a healthy weight. Patients also had the access to the Therapeutic Lymphatic Exercises module, which provided 8 avatar videos with step-by-step instructions to perform lymphatic exercises to promote lymph flow and optimize shoulder and arm mobility.

**Table 1:** The intervention strategies for the two groups.

Strategies	Interventions	
	TOLF intervention group	Arm mobility control group
**Promoting lymph flow**		
Muscle tightening deep breathing	1. At least twice a day in the morning and at night before brushing teeth or as much as the patient wants throughout the day.2. Air travel: before take-off and after landing.3. Sedentary lifestyle: At least every 4 hours.	/
Muscle tightening–pumping	1. At least twice a day in the morning and at night before brushing teeth or as much as the patient wants throughout the day.2. Air travel: before take-off and after landing.3. Sedentary lifestyle: At least every 4 hours.	/
Large muscle exercises	At least 30 minutes 3 times a week or daily.	/
**Improving arm mobility**		
Arm precaution arm mobility exercises: shoulder rolls, clasp and spread, reach to the sky, wall climb, and sideway wall stretches.	1. One week after surgery if there are no surgical drains or after the surgical drains are removed.2. At least twice a day until arm functions are returned to normal.3. Whenever arm mobility is limited throughout the recovery.	1. One week after surgery if there are no surgical drains or after the surgical drains are removed.2. At least twice a day until arm functions are returned to normal.3. Whenever arm mobility is limited throughout the recovery.
**Keeping a healthy weight**		
Eat nutrition-balanced diet (i.e., more vegetables and fruits as well as quality proteins).Maintain portion-appropriate diet (feeling75% full for each meal).	Each meal daily.	Each meal daily.
Stay hydrated	1. Drink 6-8 glasses of water daily; in the morning, before and during meals, and throughout the day.2. Avoid drinks with calories (e.g., juices).3. Drink green tea to boost metabolism.	1. Drink 6-8 glasses of water daily; in the morning, before and during meals, and throughout the day.2. Avoid drinks with calories (e.g., juices).3. Drink green tea to boost metabolism.
Get enough sleep	At least 7-8 hours of sleep per night.	At least 7-8 hours of sleep per night.

Patients assigned to the control group had the same access to the Lymphedema Knowledge module, but they had no access to the core Therapeutic Lymphatic Exercises module. Instead, they were only granted access to the Arm Mobility Exercises to promote arm mobility. The National Comprehensive Cancer Net-work (NCCN) clinical practice guidelines in oncology (27) recommended continued full use of the extremity and range-of-motion exercises (i.e., arm mobility exercises) to reduce the risk of lymphedema for all breast cancer survivors. In the current study, patients assigned to the control group had access to 4 avatar videos for improving arm mobility *via* the TOLF mHealth system.

During the first in-person research visit, the researchers spent approximately 30~45 minutes introducing the use of the TOLF program and demonstrating each module of the system to participants. It took about 10 minutes for patients in intervention group to learn the 8 avatar videos and about 5 minutes to perform a set of TOLF daily exercises each time. Participants in the control group took about 5 minutes to learn the 4 videos displaying the arm mobility exercises and it took 3 minutes to perform a set of the exercises each time. Patients were required to perform the assigned exercises at least twice a day during the 3-month study period.

### Data collection and measures

Patients’ demographic and clinical information were assessed prior to intervention at the first research visit. All outcome data were collected at baseline and at month 3 after the intervention by online questionnaires and anthropometric measurements. An evaluation module base on the mHealth system was set up. Researchers informed the participants the time of each assessment, and participants could log on to the platform to complete the online evaluation. Primary outcome of the study focused on lymphedema symptom experience measured by the Breast Cancer and Lymphedema Symptom Experience Index (BCLE-SEI); lymphedema symptom experience included the dimensions of the number of symptoms, symptom severity, and symptom distress (28). Secondary outcomes included individuals’ ADLs (measured using a subscale of the BCLE-SEI) (28) and arm volume difference (measured in-person using circumferential arm measurements) (15).

The demographic and health information were collected to include age, time since the diagnosis of breast cancer, level of education, marital status, employment status, living status, dominant hand, household incomes, affected arm, types of surgery and treatment.

The BCLE-SEI was used to measure the number of symptoms, symptom severity, symptom distress, and the impaired ADLs (28). The BCLE-SEI is a valid and reliable self-report instrument that consists of two parts, respectively evaluating the occurrence of and distress from lymphedema-related symptoms (28). Part I of the scale evaluates the occurrence (i.e., number and severity of lymphedema symptoms) of the 24 lymphedema symptoms [i.e., impaired limb mobility in shoulder, arm, elbow, wrist, and fingers, arm swelling, breast swelling, chest wall swelling, heaviness, firmness, tightness, stiffness, numbness, tenderness, pain/aching/soreness, redness, blistering, burning, stabbing, tingling (pain and needles), hotness, blistering, limb fatigue, and limb weakness]. Each symptom is rated on a Likert-type scale from 0 (no presence of a given symptom) to 4 (greatest severity of a given symptom) by symptom severity and can also be treated as a dichotomous variable with “0” indicating the absence of a given symptom, and “1”to “4” indicating the presence of a given symptom (28). Part II of the scale assesses symptom distress, that is, the negative impact and suffering evoked by an individual’s experience of lymphedema-related symptoms, including ADLs, social impact, sleep disturbance, sexuality, emotional/psychological distress, and self-perception (28). In addition to the total score of symptom distress, we also focused on the symptom distress on performing ADLs, because having difficulties in performing ADLs is an important indicator for impaired physical function and quality of life, and lymphedema symptom experience is directly associated with individuals’ ability to performing ADLs (6). The internal consistency was demonstrated with a Cronbach’s α of 0.967 for the overall BCLE-SEI; the test-retest reliability and its structure validity were also confirmed to be acceptable (29).

The arm circumferences of both arms were measured using a well-established protocol for arm circumference measurements: at 4-cm intervals consecutive measurements beginning at the wrist and ending at the shoulder to ensure accuracy (15). The arm volume was calculated using the formula *V = D (C^2^_1_ + C^2^_2_ + C_*1*_C_*2*_)/12π* where C_1_ and C_2_ represent circumferences of the two adjacent measurement locations and D is the distance between C_1_ and C_2_ (30). An interarm volume difference of ≥10% is a widely accepted diagnostic criterion for breast cancer-related lymphedema, while 5% difference in interarm volume causes symptoms and impairments in ADLs (12, 13). Therefore, this study used the interarm volume difference of ≥5% as the threshold for minimal arm volume differences.

### Sample size

Sample size calculation was based on the results (δ= 4.1, σ = 6.1) of our previous study exploring the effect of TOLF exercise on the number of lymphedema symptoms (16). The formula for comparing the difference between two means (two-sample t-test) was used: $\text{n}1=\text{n}2=\frac{{\left(Z_{\alpha }+Z_{\beta }\right)}^{2}*2\sigma^{2}}{\delta }$ and resulted in that a minimum sample size of 36 patients per group were needed with an α of 0.05 and a β of 0.8. Thus, we aimed to recruit a final sample of no less than 90 participants considering a 20% potential dropout rate.

### Statistical analysis

SPSS version 21.0 (Statistical Package for the Social Sciences; IBM Corp., Armonk, NY) was used for statistical analysis. Continuous variables were summarized using means with standard deviations (SDs) or medians with inter-quartile ranges (IQRs), depending on the variable distribution. Categorical variables were summarized using frequencies with proportions. Baseline characteristics of the intervention and control groups were compared using independent *t* test or Mann-Whitney U test for continuous variables and Chi-square test or Fisher’s exact test for categorical variables. For the study outcomes of symptom number, symptom severity, symptom distress, and ADLs, Mann-Whitney U test was performed to test the if there were any differences between the two groups. For comparing the proportion of patients presenting arm volume differences ≥5%, Fisher’s exact test was employed. In addition, generalized linear mixed-effects models were conducted to test between-group differences in the change of symptom experience, ADLs, and arm volume differences over the study period. Separate models were estimated for each outcome while adjusting for baseline characteristics that were notably different between groups and incorporated fixed effects for time (baseline vs. 3-month), treatment group (intervention vs. control), and a time by group interaction term, as well as random effects for individuals to account for the repeated measures. The level of statistical significance was set at 0.05, and all statistical tests were 2-sided.

## Results

### Study participants

**Figure 1:**
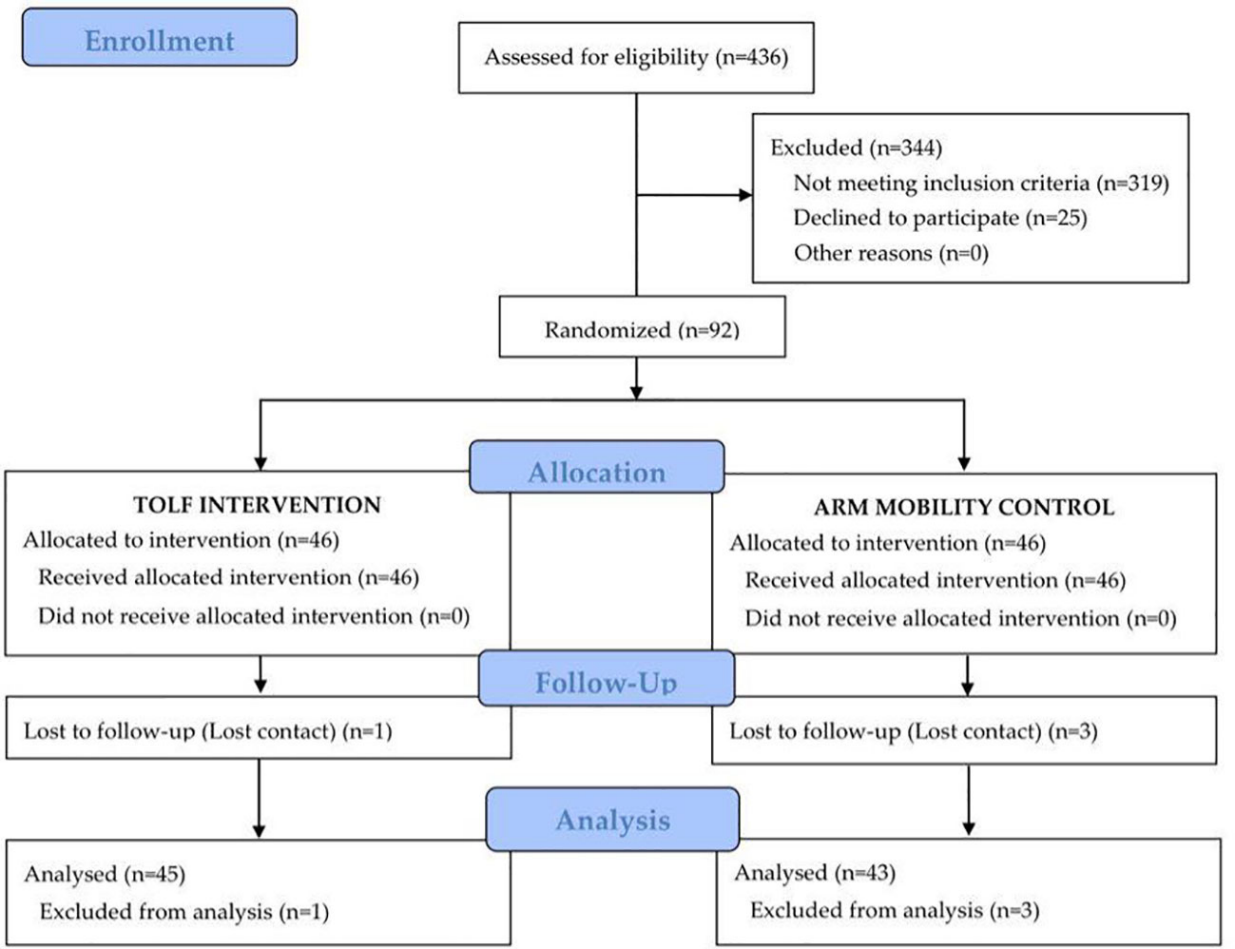
Participant flow chart.

During the recruitment period, 436 patients were assessed for eligibility, 92 met the eligibility criteria and were randomly assigned at a 1:1 ratio to either the TOLF intervention group or the arm mobility control group. All participants learned to use the mHealth system within 45 minutes and were able to perform lymphatic exercises or arm mobility exercises using the avatar videos based on the assigned treatment groups. At the end of the 3-month intervention, one patient in the intervention group and 3 patients in the control group were lost to follow-up. The participant flowchart is presented in **Figure 1**. Baseline characteristics of all participants are shown in **Table 2**. There were no significant differences in the baseline characteristics between the two groups except for the affected arm (*P=*0.001).

**Table 2:** Demographic and clinical characteristics of participants at baseline (N=92).

Characteristics	Total(N=92)	TOLF group(n=46)	Arm mobility group (n=46)	Statistics (*df*)	*P*-value
**Age (years), mean (SD)**	47.23±7.74	46.91±8.31	47.54±7.20	t_90_=0.389	0.698
**Time since diagnosis (months),** **mean (SD)**	15.88±12.35	16.76±11.51	15.00±13.21	t_90_=-0.682	0.497
**Level of education, n (%)**	** **	** **	** **	Fisher’s exact test (4)	0.644
Primary school or below	8 (8.7)	3 (6.5)	5 (10.9)		
Middle school	22 (23.9)	10 (21.7)	12 (26.1)		
High school	19 (20.7)	10 (21.7)	9 (19.6)		
Associate degree	17 (18.5)	7 (15.2)	10 (21.7)		
Bachelor’s degree or above	26 (28.3)	16 (34.8)	10 (21.7)		
**Marital status, n (%)**	** **	** **	** **	𝑥_1_^2^<0.001	>0.999
Married	86 (93.5)	43 (93.5)	43 (93.5)		
Single/divorced	6 (6.5)	3 (6.5)	3 (6.5)		
**Employment status, n (%)**	** **	** **	** **	𝑥_1_^2^<0.011	>0.999
Unemployed	46 (50.0)	23 (50.0)	23 (50.0)		
Employed	46 (50.0)	23 (50.0)	23 (50.0)		
**Living status, n (%)**	** **	** **	** **	𝑥_1_^2^<0.001	>0.999
Live alone	3 (3.3)	2 (4.3)	1(2.2)		
Live with family	89 (96.7)	44 (95.7)	45(97.8)		
**Dominant hand, n (%)**	** **	** **	** **	𝑥_1_^2^<0.001	>0.999
Left	7 (7.6)	4 (8.7)	3 (6.5)		
Right	85 (92.4)	42 (91.3)	43 (93.5)		
**Perceived household incomes, n (%)**				Fisher’s exact test (2)	0.235
Do not have enough to make ends meet	20 (21.7)	12 (26.1)	8 (17.4)		
Have enough to make ends meet	60 (65.2)	26 (56.5)	34 (73.9)		
Comfortable: have more than enough to make ends meet	12 (13.0)	8 (17.4)	4 (8.7)		
**Affected arm, n (%)**				𝑥_1_^2^=7.351	0.001
Left	45 (48.9)	29 (63.0)	16 (34.8)		
Right	47 (51.1)	17 (37.0)	30 (65.2)		
**Types of surgery, n (%)**				𝑥_1_^2^=0.178	0.677
Mastectomy Lumpectomy	86 (93.5) 6 (6.5)	44 (95.7) 2 (4.3)	42 (91.3) 4 (8.7)		
**Axillary lymph node dissection, n (%)**				𝑥_1_^2^=0.052	>0.999
Yes	65 (70.7)	32 (69.6)	33 (71.7)		
No	27 (29.3)	14 (30.4)	13 (28.3)		
**Sentinel lymph nodes biopsy alone, n (%)**				𝑥_1_^2^=0.256	0.801
Yes	20 (21.7)	11 (23.9)	9 (19.6)		
No	72 (78.3)	35 (76.1)	37 (80.4)		
**Chemotherapy, n (%)**				𝑥_1_^2^=1.022	0.449
Yes	72 (78.3)	34 (73.9)	38 (82.6)		
No	20 (21.7)	12 (26.1)	8 (17.4)		
**Radiotherapy, n (%)**				𝑥_1_^2^<0.001	>0.999
Yes	3 (3.3)	2 (4.3)	1 (2.2)		
No	89 (96.7)	44 (95.7)	45 (97.8)		

TOLF, The-Optimal-Lymph-Flow; SD, standard deviation; df, degree of freedom.

### Symptom experience and ADLs

At baseline, there were no significant differences in terms of the median scores of the number of symptoms (*P=*0.271), symptom severity (*P=*0.117), symptom distress (*P=*0.710), or ADLs (*P=*0.706) between the two groups (**Table 3**). At the endpoint of the 3-month intervention, the TOLF group had significantly lower median scores for the number of symptoms (Med_TOLF_=5.00, IQR=2.00-7.00 vs Med_Arm Mobility_=10.00, IQR=6.00-14.00; *P<*0.001), symptom severity (Med_TOLF_=5.00, IQR=2.00-7.00 vs Med_Arm Mobility_=12.00, IQR=6.00-17.00; *P<*0.001) and ADLs (Med_TOLF_=4.00, IQR=4.00-6.00 vs Med_Arm Mobility_=6.00, IQR=4.00-9.00; *P=*0.001).

**Table 3:** Comparison of symptom experience and ADLs between the TOLF intervention group and arm mobility control group at baseline and study endpoint.

Outcomes	TOLF group Median (IQR)	Arm mobility group Median (IQR)	Test for between-group differences		
**Baseline**	n=46	n=46	***Wilcoxon’s r*** **(95% CI)** ^**a**^	***W-score***	***P-*** **value**
Number of symptoms	11.00 (7.75-15.25)	12.00 (10.00-15.25)	0.114 (-0.103 to 0.305)	1998.5	0.271
Symptom severity	14.00 (10.00-22.00)	18.00 (12.75-22.25)	0.164 (-0.065 to 0.343)	1938.5	0.117
Symptom distress	24.00 (17.75-32.50)	23.50(18.00-29.25)	0.032 (-0.173 to 0.235)	2186.5	0.710
ADLs	12.00 (9.00-13.25)	12.00 (9.75-14.00)	0.045 (-0.161 to 0.247)	2091.0	0.706
**3-month follow-up**	**n=45**	**n=43**	***Wilcoxon’s r*** **(95% CI)** ^**a**^	***W-score***	***P*** **-value**
Number of symptoms	5.00(2.00-7.00)	10.00(6.00-14.00)	0.457(0.099 to 0.517)	1534.5	<0.001
Symptom severity	5.00(2.00-7.00)	12.00(6.00-17.00)	0.402 (0.072 to 0.490)	1584.0	<0.001
Symptom distress	14.00(9.00-18.00)	14.00(9.00-24.00)	0.055 (-0.157 to 0.261)	1942.5	0.616
ADLs	4.00(4.00-6.00)	6.00(4.00-9.00)	0.352 (0.046 to 0.464)	1629.5	0.001

TOLF, The-Optimal-Lymph-Flow; IQR, inter-quartile range; CI, confidence interval; ADLs, activities of daily living.

^a^Wilcoxon’s r: Measure of effect size, recommended interpretation: 0.1=small, 0.3=medium, 0.5=large.

Generalized linear mixed-effects models were used to predict the symptom experience (number of symptoms, symptom severity, and symptom distress) and ADLs across the two measurement time points and to determine group differences in the changes over the study. As shown in **Table 4**, there was a significant improvement in symptom experience and ADLs for both groups from baseline to the endpoint. There was no significant time by group interaction effect across all study outcomes. In addition, significant between-group differences in the changes of number of symptoms (*P<*0.001) and symptom severity (*P=*0.012) throughout the course of the study were detected.

**Table 4:** Results of the generalized linear mixed-effects models for symptom experience and ADLs.

Outcomes	Number of symptoms^a^		Symptom severity^a^		Symptom distress^a^		ADLs^a^	
	Estimates(95% CI)	*P-*value	Estimates(95% CI)	*P-*value	Estimates(95% CI)	*P-*value	Estimates(95% CI)	*P-*value
Intercept	6.09(4.13 to 8.06)	<0.001	8.46(5.46 to 11.47)	<0.001	18.21(14.30 to 22.13)	<0.001	6.80(4.91 to 8.69)	<0.001
Group	4.23(1.76 to 6.71)	<0.001	4.77(1.07 to 8.46)	0.012	0.57(-4.38 to 5.53)	0.819	1.46(-0.99 to 3.90)	0.240
Time	5.50(3.32 to 7.67)	<0.001	8.02(4.50 to 11.54)	<0.001	10.51(6.19 to 14.82)	<0.001	5.43(3.41 to 7.45)	<0.001
Group×Time	-2.72(-5.81 to 0.38)	0.085	-1.24(-6.25 to 3.76)	0.624	-3.55(-9.69 to 2.60)	0.256	-1.39(-4.27 to 1.49)	0.342

ADLs, activities of daily living; CI, confidence interval.

^a^The models were adjusted for the baseline characteristics of “affected arm”, which was notably different between groups.

### Arm volume differences

At baseline prior to the intervention, there was no significant difference between the two groups in terms of ≥5% arm volume differences (**Table 5**). At the study endpoint of 3 months, the TOLF group had significantly fewer participants with ≥5% arm volume differences (11.1% [5/45] vs 30.2% [13/43], *P=*0.035). There was a 12.8% reduction (from 23.9% [11/46] to 11.1% [5/45]) in the proportion of patients with ≥5% arm volume differences from baseline to post-intervention in the TOLF group, while there was a 1.9% increase (from 28.3% [13/46] to 30.2% [13/43]) in the proportion of patients with ≥5% arm volume differences in the arm mobility group. Moreover, the proportion of new cases with ≥5% arm volume differences in the arm mobility group was larger than that in the TOLF group (20.0% [6/30] vs 5.9% [2/34]; **Table 5**).

**Table 5:** Comparison of arm volume differences between the TOLF intervention group and arm mobility control group at baseline and study endpoint.

Outcomes	TOLF group N (%)		Arm mobility group N (%)		Fisher’s exact test of independence	
**Baseline**	**n=46**		**n=46**		**Odds ratio (95% CI)^a^**	***P*-value**
	**Yes**	**No**	**Yes**	**No**		
Arm volume difference > 5%	11 (23.9)	35 (76.1)	13 (28.3)	33 (71.7)	1.12 (0.69 to 1.84)	0.811
**3-month follow-up**	**n=45**		**n=43**		**Odds ratio (95% CI)^a^**	***P*-value**
	**Yes**	**No**	**Yes**	**No**
Arm volume difference > 5%	5 (11.1)	40 (88.9)	13 (30.2)	30 (69.8)	2.06 (0.95 to 4.45)	0.035

TOLF, The-Optimal-Lymph-Flow; CI, confidence interval. ^a^Odds ratio: a measure of effect size, recommended interpretation: 1.5=small, 2=medium, 3=large.

As shown in **Table 6**, there were significant differences between the two groups throughout the course of the study in changes of arm volume differences ≥5% (*P*=0.034). There was no significant time by group interaction effect (*P*=0.209, *P*=0.868).

**Table 6:** Results of the binomial generalized linear mixed-effects models with a logic link function for arm volume differences.

Outcomes	Arm volume difference ≥5%^a^	
	Odds ratios (95% CI)^b^	*P-*value
Intercept	1.77 (0.77 to 2.78)	0.001
Group	-1.28 (-2.47 to -0.10)	0.034
Time	-0.97 (-2.15 to 0.22)	0.109
Group×Time	0.97 (-0.55 to 2.48)	0.209

CI, confidence interval.

^a^The models were adjusted for the baseline characteristics of “affected arm”, which was notably different between groups. ^b^Odds ratio: a measure of effect size, recommended interpretation: 1.5=small, 2=medium, 3=large.

## Discussion

A total of 88 at-risk breast cancer patients have completed the 3-month intervention using the TOLF mHealth system in the current study. Significant benefits of the early postoperative TOLF intervention for improving lymphedema symptom experience and optimizing arm volume status were identified. Prior studies have recognized the TOLF intervention as a safe, feasible, and efficacious replacement or complement therapy to manage lymphedema symptoms for breast cancer survivors (16, 18, 25). Extending previous research findings, the present RCT further confirmed the preventive effects of TOLF program in breast cancer survivors who are at risk of developing lymphedema in the early postoperative period (1-month post-surgery).

The study findings revealed that the number and the severity of lymphedema symptoms as well as the impact of these symptoms on patients’ ADLs have been significantly improved by the TOLF intervention in comparison with arm mobility exercise control. Meanwhile, participants in both groups showed improvements in symptom experience and ADLs over the 3 months, whereas those received the TOLF intervention gained greater benefits in reducing the number and severity of lymphedema symptoms. The results suggest that TOLF program should be a promising choice for lymphedema symptom management and prevention of subclinical lymphedema progression. Compared with arm mobility exercises, the TOLF intervention added a set of exercises that were designed to activate lymphatic system involving muscle-tightening deep breathing, muscle-tightening pumping, and large muscle exercises. The muscle-tightening deep breathing and muscle-tightening pumping exercises combine muscle tightening, stretching and pumping movements coordinated with synchronized deep breathing to mimic the physiological process of lymph propulsion, which could render a synergistic effect to stimulate lymph fluid removal in both the affected arm as well as the whole body (19). Large muscle exercises such as walking, jogging and bicycling induce not only musculoskeletal contractions, but also breathing alterations, arterial pulsations, skin tensions, and postural changes (31, 32). These physiological adaptions also help to facilitate lymphatic systems and promote lymph flow throughout the overall body (19, 25). As a result, the therapeutic lymphatic exercises could benefit patients in alleviating lymph fluid accumulation-related symptoms and preventing subclinical lymphedema. On the other side, the set of exercises gradually contribute to relieving the armpit and chest wall tightness caused by the adhesion of the pectoralis major and axillary tissue after surgically incised skin wounds sealed, promoting the formation of loose connective tissue and decreasing tethering of scar tissue. Thus, the exercises also help to reduce distressing symptoms that are related to the impaired arm mobility caused by scar adherence from operative incisions (19).

Findings of our study demonstrated that the TOLF intervention was effective in decreasing lymph fluid levels, indicated by the changes in arm volume differences over the study period. It is important to note that a 12.8% reduction in the proportion of patients with ≥5% arm volume differences from baseline to postintervention was observed in the TOLF intervention group, whereas a 1.9% increase in the arm mobility control group was detected in this study. Moreover, the proportion of new cases with ≥5% arm volume differences in the arm mobility group was higher than that in the TOLF group (20.0% vs 5.9%). Our findings are consistent with a previous study (19), which revealed that 97% of 134 breast cancer patients who received TOLF lymphatic exercises maintained or decreased their preoperative arm volumes at 12 months after surgery, and there was a 12% reduction in proportions of patients with ≥5% arm volume differences in the TOLF group (17). The findings suggest that the TOLF intervention should be more effective in reducing arm volume than the arm mobility control. Muscle-tightening deep breathing and muscle-tightening pumping performed by patients in the TOLF group could facilitate lymph fluid flow and drain *via* repeated arm muscle tightening, stretching, and pumping movements. Combined with large muscle exercises, the TOLF intervention helps to stimulate the whole-body lymphatic system function more efficiently and further enhance the muscle milking and pumping actions (16). Taken together, application of TOLF lymphatic exercises for breast cancer survivors at risk of developing lymphedema should be effective in preventing or reversing the subclinical stage of breast cancer-related lymphedema and avoiding the increase in arm circumference and volume.

To our knowledge, this is the first study that examined the preventive effects of the TOLF intervention among breast cancer survivors at risk of developing lymphedema in the early postoperative period. Managing lymphedema symptoms is critical to reduce the risk of lymphedema. Breast cancer survivors who report more lymphedema symptoms are more likely to eventually develop lymphedema (21). TOLF intervention focusing on self-management risk reduction strategies in activating lymphatic system to manage lymphedema symptoms, shows great promise for prevention of the progression of latent lymphedema and this intervention holds a number of strengths. First, the low-cost and technologically driven delivery model of the TOLF intervention make it relatively easy to implement and dissemination in clinical practice or at home. Second, the web- and mobile-based TOLF system offered clear instructions about how exercise should be done and how often it should be done to ensure that patients would like to initiate and adhere to the prescribed therapeutic exercise regimen. Third, the TOLF intervention consists of a series of relatively low-intensity exercises and requires low physical demands, as thus the intervention adds little burden on patients and can be appropriate for these vulnerable survivors who underwent surgical operations just one month ago. Finally, the underlying premise of TOLF program is to empower, rather than inhibit, how breast cancer survivors live their lives by emphasizing “what to do,” rather than “what to avoid.” Therefore, the TOLF intervention can be thought as a pragmatic, accessible, acceptable, and well-tolerated self-care strategy for the management and prevention of lymphedema and associated symptoms.

Although our sample size had adequate power for the trial, the comparatively small sample size and single-site trial are limitations of this study. Another limitation of our trial lies in the lack of real-time monitoring of exercise implementation and quality. Future research that includes a larger sample and takes advantage of wearable devices to support real-time monitoring is warranted to verify our study findings. A strength of the study is the comparison of the TOLF intervention versus arm mobility control, which provides evidence for optimal exercise decision-making in breast cancer survivors in the early postoperative period. Meanwhile, the use of technologically driven digital therapy not only enhanced the fidelity, transparency, and reproducibility of the intervention but also improved the patients’ ability to learn and perform the assigned exercise therapy given.

In conclusion, the results of this RCT showed significant benefits of the early postoperative TOLF intervention for managing lymphedema symptoms, improving ADLs, and optimizing arm volume status among breast cancer survivors at risk of developing lymphedema. These findings suggest that the TOLF intervention should be considered as a non-pharmacological, educational, and behavioural strategy for lymphedema prevention and lymphedema symptom management in the early postoperative period.

## Data availability statement

The original contributions presented in the study are included in the article/**Supplementary Material**. Further inquiries can be directed to the corresponding authors.

## Ethics statement

This study was approved by the Biomedical Ethics Committee of the West China Hospital, Sichuan University (Approval number: 2019/24). The patients/participants provided their written informed consent to participate in this study.

## Author contributions

Conceptualization: MRF, XF, and XD. Methodology: MRF, XF, YL and XD. Intervention: XD, YS and AZ. Investigation: XD, LF, HC, XZ, YS and AZ. Data curation: XD, YS and AZ. Writing—original draft preparation: XD and YL. Writing—review and editing: MRF, YL and XF. Supervision: XF and MF. Funding acquisition: XF.

## Funding

This research was funded by Sichuan Province Science and Technology Development Funds (grant number 2019YFS0295).

## Acknowledgments

We thank all the patients who participated in the study.

## Conflict of interest

The authors declare that the research was conducted in the absence of any commercial or financial relationships that could be construed as a potential conflict of interest.

## Publisher’s note

All claims expressed in this article are solely those of the authors and do not necessarily represent those of their affiliated organizations, or those of the publisher, the editors and the reviewers. Any product that may be evaluated in this article, or claim that may be made by its manufacturer, is not guaranteed or endorsed by the publisher.
